# Fumonisins, Trichothecenes and Zearalenone in Cereals

**DOI:** 10.3390/ijms9112062

**Published:** 2008-10-31

**Authors:** Selma Yazar, Gülden Z. Omurtag

**Affiliations:** Department of Pharmaceutical Toxicology, Faculty of Pharmacy, Marmara University, 34668, Haydarpaşa - İstanbul, Turkey. E-Mail: selmayazar@hotmail.com

**Keywords:** Fumonisins, trichothecenes, zearalenone, cereals

## Abstract

Fumonisins are phytotoxic mycotoxins which are synthesized by various species of the fungal genus *Fusarium* such as *Fusarium verticillioides* (Sacc.) Nirenberg (ex *F.moniliforme* Sheldon) and *Fusarium proliferatum*. The trichothecene (TC) mycotoxins are secondary metabolites produce by species that belong to several fungal genera, especially *Fusarium*, *Stachybotrys*, *Trichothecium*, *Trichoderma*, *Memnoniella* and *Myrothecium. Fusarium* mycotoxins are widely dispersed in cereals and their products. Zearalenone (ZEA) is an estrogenic compound produced by *Fusarium* spp. such as *F. graminearum* and *F. culmorum*. Fumonisins, the TCs and ZEA are hazardous for human and animal health. Contamination with TCs causes a number of illnesses in human and animal such as decrease in food consumption (anorexia), depression or inhibition on immune system function and haematoxicity. The purpose of this paper is to give a review of the papers published on the field of fumonisin, TC and ZEA mycotoxins in cereals consumed in the world.

## 1. Introduction

Mycotoxins are secondary metabolites produced by a wide variety of fungal species that cause nutritional losses and represent a significant hazard to the food chain [1]. The exposure risk to human is either directly through foods of plant origin (cereal grains) or indirectly through foods of animal origin (kidney, liver, milk and eggs) [2–4]. *Fusarium* species are probably the most prevalent toxin-producing fungi of the northern temperate regions and are commonly found on cereals grown in the temperate regions of America, Europe and Asia [5].

The most important *Fusarium* mycotoxins are fumonisins, TCs such as T-2, HT-2, DON, DAS, FUS-X, NIV, diacetylnivalenol, neosolaniol and ZEA. They are common mycotoxins throughout the world, mainly associated with cereal crops, in particular corn, wheat, barley, rye, rice and oats [6–9]. The summary of the IARC evaluation of *Fusarium* mycotoxins [10] is reported in Table 1.

## 2. Fumonisins

### 

#### Short history and synthesis

Elucidation of the chemical structure of fumonisins was first clarified by Bezuidenhout *et al.* [11]. Fumonisins are produced by a number of *Fusarium* species, notably *F. verticillioides* (formerly *Fusarium moniliforme*=*Gibberella fujikuroi*), *F. proliferatum*, *F. anthophilum*, *F. nygamai* as well as *Alternia alternata* f. sp. l*ycopersici* [12]. Until now, twenty-eight fumonisins have been isolated and they can be divided in four series known as A, B, C and P. FB_1_, FB_2_ and FB_3_ are the principal fumonisins analyzed as natural contaminants of cereals [13, 14]. *F. verticillioides* produce several mycotoxins, the most prominent of which is called fumonisin B_1_ (FB_1_) [15]. The carcinogenic mycotoxin fumonisin B_2_ was discovered for the first time in *Aspergillus niger*, an industrially important species [16].

FB_1_ is the diester of propane-1,2,3-tricarboxylic acid and a pentahydroxyeicosane in which the C14 and C15 hydroxy groups are esterified with the terminal carboxy group of propane-1,2,3-tricarboxylic acid (TCA). FB_2_ is the C-10-deoxy analogue of FB_1_ and FB_3_ is the C-5-deoxy analogue of FB_1_ [13]. *Fusarium* mycotoxins found in food are produced mainly in the field, although some toxin synthesis may occur during storage. Temperature and moisture conditions are crucial factors affecting fungal infection and toxin synthesis [17]. Infection of cereal grains with *Fusarium* species can trigger serious human and animal diseases [8, 18]. *F. verticillioides* was the predominant fungus isolated from moldy corn associated with a field outbreak of equine leukoencephalomalacia (ELEM) in South Africa during 1970 characterized by liquefaction’s necrosis in the white matter of the cerebral hemispheres of horses [19]. Fumonisin toxicosis in swine was mentioned porcine pulmonary edema (PPE) after outbreaks of a fatal disease in pigs fed with *F. verticillioides* contaminated corn screenings from the 1989 corn crop in Iowa, Illinois, and Georgia [20].

#### Stability

Castelo *et al*. [21] have reported that fumonisins added to cornmeal and present in artificially contaminated cornmeal samples were unstable under roasting conditions, but remained fairly stable during canning and baking of corn-based foods because canned and baked products reached lower temperatures than the roasted products. Alberts *et al.* [22] suggested that FB_1_ was found to be stable to heating, as there was no reduction in the FB_1_ level after boiling culture material of *F. moniliforme MRC 826.* Jackson *et al.* [23] indicated that foods reaching temperatures greater than 150 °C during processing may have lower fumonisin levels. Marin *et al.* [24] showed that water activities (a_w_, 0.968, 0.956, 0.944, 0.925) and temperature (25 °C and 30 °C) affected colonization and production of FB_1_ and FB_2_. They suggested at all a_w_ levels and both temperatures there was an increase in FB_1_ and FB_2_ concentration with time.

#### Products

In addition to corn or corn-based foods and feeds [25, 26], the occurrence of fumonisins has also been reported some products such as beans, rice, sorghum, corn, wheat noodles, curry, chili pickle, beer and corn-based brewing adjuncts [27]. Between 1997 and 2002, the studies related to fumonisin in cereals have been reported by Soriano and Dragacci [28]. The fumonisin studies on cereals between 2003–2007 are shown in Table 2.

#### Mode of action

Fumonisins structurally resemble sphingoid bases such as sphingosine. The structural similarity between sphinganine and FB_1_ suggests that the mechanism of action of this mycotoxin is mainly via disruption of sphingolipid metabolism. This mechanism is reflected in effects on protein kinase activity, on cell growth and differentiation, in cell death (apoptosis), carcinogenicity and involvement of lipid peroxidation. Inhibition of biosynthesis of sphingolipids has seen at different levels and is reflected in changes of the ratio sphinganine/sphingosine (Sa/So). This ratio may be used as indicators of FB_1_ exposure, mechanism of action of sphingolipids and their metabolites in the toxicity of FB_1_ is well documented by Soriano *et al.* [13]. Structurally, fumonisins resemble sphingolipids and can alter sphingolipid biosynthesis suggesting that sphingolipid alterations play an important role in disease and carcinogenesis in DNA damage for FB_1_ [15, 29].

#### Toxicity

Fumonisins are known to be the cause of leukoencephalomacia in equines [30–33] and in rabbits [34], pulmonary edema and hydrothorax in swine [34, 35], cardiac failure in baboons [36], atherogenic effects in vervet monkeys [37], brain haemorrhage in rabbits [38], renal cancer and hepatocarcinogenic in rats [39–41] and some birth defects (especially neural tube defects) [42]. Fumonisins additionally produce mild to fatal toxicity in liver, kidney and heart in horses, pigs, cattle, sheep, chickens, ducks, rabbits, rats and mice [34, 40, 43–45]. The effects of cytotoxicity of FB was observed in turkey and in broiler chicks lymphocytes [46, 47], chick macrophages [48], in rabbit kidney RK13 cells [49]. Epidemiological evidence indicates a link between human esophageal cancer and ingestion of *Fusarium verticillioides*-contaminated corn [30]. FB_1_, in cereals was associated with the incidence of a high rate of human esophageal cancer in Africa [13, 31], in northern Italy [50], in Iran [51], the Southeastern of the United States [2, 52] and with promotion of primary liver cancer in certain endemic areas of the People’s Republic of China [53, 54]. There are no confirmed biomarkers for human exposure to FB_1_ [30].

The International Agency for Research on Cancer (IARC) has evaluated the cancer risk of fumonisin to humans and grouped them as group 2B (probably carcinogenic). They are toxic to animals and at least one analogue, FB_1_, is carcinogenic to rodents. Their effect on human health is unclear. The mechanisms of FB_1_-induced carcinogenesis are uncertain and the information on FB_1_ mutagenic properties is limited and controversial [15, 40, 45, 55–60]. Some reports show that fumonisins have been described some genotoxic effect in mammalian cells *in vitro*, including clastogenic effects, chromosomal aberrations and sister chromatid exchange, or DNA synthesis [30].

In some animal species such as horse, mouse, pig and rat; NOAELs expressed as mg FB_1_/kg body weight [13]. The recommended maximum levels for fumonisins in corn and corn products intended for human consumption (Table 3) are based on concerns associated with hazards show primarily by animal studies. However, based on available information on the occurrence of fumonisins, FDA accepted that typical fumonisin levels found in corn and corn products intended for human consumption are much lower than the recommended levels [61].

A provisional maximum is fixed for tolerable daily intake (PMTDI) for FB_1_, B_2_ and B_3_ single or in combination, of 2 μg/kg of body weight per day on the basis of the NOEL of 0.2 mg/kg of body weight per day and safety factor of 100. Some national estimates of intake of FB_1_ in Europe and in the world have displayed Table 4 [5].

## 3. Trichothecenes

### 

#### Short history and synthesis

TCs are mycotoxins produced by a range of different fungi and chemically they belong to the sesquiterpenoids [62]. TCs have been suspected to produce a human illness known a “taumelge treide” (staggering grains) that was first observed in Siberia in the 1890s. Symptoms in this included vomiting, headache, and vertigo [63]. A possible role of TCs in the human disease Alimentary Toxic Aleukia (ATA) in Russia was reported. It has been reported since the 19^th^ century and a severe outbreak occurred in the Orenburg region where 100,000 people died [64]. Later, TCs have also been researched cause of concerns about their potential misuse as biological or chemical warfare agents. In late 1970s and early 1980s, it was asserted that weaponized, aerosolized TCs had been used on civilian and refugee populations in Laos, Kampuchea, and Afghanistan. It was reported symptoms such as bleeding, nausea, fever, dyspnea, dizziness, and vertigo. These events became known as "yellow rain", because of descriptive reports of witnesses, although the allegation that TCs were responsible for the reported symptoms is controversial [65]. The levels and kinds of TCs were in positive correlation with the incidence of esophageal cancer in human [66]. Trichothecin was isolated first TC, was isolated from *Trichothecium roseum* and described in 1949 by Freeman and Morrison. Following the isolation of trichothecin was followed by the isolation and description of other TCs such as DAS, T-2, NIV and DON. By now, more than 180 different trichothecenes and trichothecene derivatives have been isolated and characterized [64, 67]. The TCs causing most concern are T-2, which is the most acute toxic TC, HT-2, and NIV [67].

The TCs are all non-volatile, low-molecular-weight sesquiterpene epoxides, and can be further classified according to the presence or absence of characteristic functional groups. The C12,13-epoxide ring, which is necessary for protein synthesis inhibition, is considered to be essential [63–65]. Most TCs also have a C9–10 double bond, which is important for their toxicity [64, 68].

They can be divided into four categories according to both their chemical properties and their producer fungi;

1. **Type A:** functional group other than a ketone at C8 position (e.g.; T-2, HT-2, DAS);
2. **Type B:** carbonyl functions at C8 position (e.g.; DON, NIV, FUS-X, 3-acetyl-deoxynivalenol, 15-acetyl-deoxynivalenol);
3. **Type C:** second epoxide group at C7, 8 or C9, 10; (e.g.; crotocin and baccharin);
4. **Type D:** macrocyclic ring system between C4 and C15 with two ester linkages (e.g.; satratoxin G, H, roridin A and verrucarin A) [65, 68–70].

#### Stability

TCs are stable to heating and are not degraded during normal food processing or autoclaving. They are also stable at neutral and acidic pH and consequently, they are not hydrolyzed in the stomach after ingestion [68]. The degree of infection is dependent on various factors, for example weather conditions, environmental conditions, and temperature and storage conditions of cereal crops [63, 65]. They can be deactivated under strong acid and alkaline conditions [65]. At harvest and storage of cereals, TCs formation is the key factors such as conidia presence and humidity combined with temperature. Minimizing or avoiding conidia contaminated materials, cleaning at early stage during the harvest and drying the grain at low temperatures will allow to store cereals for more than 12 months without increasing TC levels [71]. *Fusaria* are common fungi on a variety of plants and soil throughout the cold and cold-temperature regions about 0–10 °C and they necessary moisture in range of 22–25%. The effect of solvent, storage, temperature, moisture, pH and heat on the stability of TCs was documented by Widestrand and Pettersson [72] and Lauren and Smith [73].

#### Products

They are commonly found on cereals grown in the temperate regions of Europe, America and Asia. The toxin is probably the most important of the northern temperate regions [63, 74]. The toxin is commonly found world-wide on cereals such as wheat, rye, barley, oats and corn. Cereals products are prevalent used in feed and farm animals may thus consume relatively high amounts TCs [64, 67]. The TCs studies in cereals between 1999–2008 are shown in Table 5.

#### Mode of action

TCs are especially well-known inhibitors of protein synthesis, including DNA, RNA synthesis, inhibition of mitochondrial function, effects on cell division and membrane effects. The toxin binds to the peptidyl transferase, which is an integral part of the 60S ribosomal subunit of mammalian ribosome. The mechanism of DNA synthesis has not yet been clarified, however, it may be a secondary effect of the inhibition of the protein synthesis or of the apoptotic effect of TCs. TCs affect cell membrane and are also shown to interfere with the metabolism of membrane phospholipids and to increase liver lipid peroxides *in vivo*. Also, some TCs are shown to change the serotonin activity in the central nervous system, which is known to be related in the regulation of food intake [62, 67].

#### Toxicity

General signs of TCs toxicity in animals include weight loss, decreased feed conversion, feed refusal, vomiting, bloody diarrhea, severe dermatitis, hemorrhage, decreased egg production, abortion, and death. Clinical effects produced by TCs can be grouped into four clinical categories: (1) feed refusal, (2) dermal necrosis, (3) gastroenteric effects, (4) coagulopathy [75]. TC exposure leads to apoptosis both *in vitro* and *in vivo* in several organs such as lymphoid organs, hematopoietic tissues, liver, intestinal crypts, bone marrow and thymus [64, 68, 70]. Acute high dose toxicity of TCs is characterized by “radiomimetic” effects such as diarrhea, vomiting, leukocytosis, haemorrhage, and circulatory shock and death, whereas chronic low dose toxicity is described by anorexia, reduced weight gain, decreased feed conversion, neuroendocrine changes and immunologic effects [62, 70, 74]. Cellular effects on DNA and membrane integrity have been considered as secondary effects of the inhibited protein synthesis [62, 67]. Cytotoxic effects were observed at slightly higher doses of TCs [66]. The myelotoxicity was considered highest for T-2 and HT-2 toxins and lowest for DON and NIV [74].

TCs cause the greatest problems to animal health [62]. In acute tests with TCs, type A members such as DAS and T-2 have been found to be more toxic than Type B members such as DON and NIV [76]. TCs are toxic to many animal species, but the sensitivity varies considerably between species and also between the different TCs. Chickens are more sensitive to TCs than ruminants. Turkeys are more sensitive to TCs than chickens. Pigs are especially sensitive to TCs in feed [64]. TCs reason histological finding in experimental animals such as cellular necrosis and karyorrhexis in actively dividing tissues of the intestinal mucosa, bone marrow, spleen, testis, and ovary [62].

There is very little data about toxicokinetics of TCs in humans. It is unknown whether the metabolic and elimination pathways that have been described in these studies are predictive of TC toxicokinetics in humans. Further research is necessary to understand the human health effect of TCs from dietary as well as non-dietary routes of exposure. The majority of the studies have been assigned in animals, where interspecies variation in pharmacokinetic parameters and susceptibility to TCs has been constantly reported. [65]. In a study of cultured human lymphocytes, increased Ig production was observed in cells exposed to lower levels of TCs (T-2, DAS, DON, NIV), whereas decreased Ig production was reported at higher levels [77]. TCs were observed in combine with ATA led some researchers to evaluate whether they may have a beneficial role in the treatment of cancer [65].

### 3.1. T-2 and HT-2

#### Short history and synthesis

The first report of ATA happened in Russia during World War II and the other was the bean-hull poisoning of horses in Japan. In both event, isolates of *F. sporotrichioides* isolated from grains and beans were found to produce T-2 and its derivatives [78]. Generally, HT-2 occurs together with T-2 in the infected cereals products. HT-2 is a major metabolite of T-2 and T-2 is only differentiated from HT-2 by an acetyl group in the C-4 position. It is difficult to distinguish the effects of T-2 and HT-2 *in vivo* because of HT-2 is rapidly formed after exposing an animal to T-2. [67]. T-2 and HT-2 have been reported to be produced by *F. sporotrichioides*, *F. poae*, *F. equiseti* and *F. acuminatum* [5]. T-2 is a secondary metabolite produced by *Fusarium* and related fungal species [79]. *F. sporotrichioides* of the Sporotrichiella section is the most important producer of T-2. This species has no known teleomorph. It is basically a saprophytic species (non pathogenic to plants) and is especially associated with cereals left in the field after normal harvest [74].

#### Stability

Mainly factors of occurring of *F. sporotrichioides* in cereals are a result of water damage to grains occurring when the cereals remain for extended periods on the field at or after harvest or when the grain is wet during storage [64]. *F. sporotrichioides* grows at −2 to 35 °C and only at high water activities [5]. The optimum temperature for occur of T-2 is relatively low (8–14 °C), with yields being much lower or negligible at temperatures of 25 °C and above [80]. T-2 is produced by various species of *Fusaria* which are widespread fungi on a variety of plants in soil throughout the cold-temperate regions [81]. T-2 has the most stability at 4 °C and the least stability at 37 °C [82]. T-2 and HT-2 have the most stability when stored at −70 °C, in the presence of NaF, and in urine (pH 6). T-2, HT-2 and T-2 tetraol in urine and blood stored over 6 months at −70, 4, and 23 °C with and without the addition of NaF. They have less stability in saline (control, pH 7) and the least stablity in blood (pH 8). Since T-2 tetraol is the most stability metabolite and, it is the most appropriate metabolite for diagnostic testing [83].

#### Products

T-2 and HT-2 are generally found in various cereal crops such as wheat, corn, barley, oats and rye and processed grains (malt, beer and bread) [84].

#### Mode of action

T–2 is well known to inhibit DNA, RNA and protein synthesis, mitochondrial function as well as other subcellular processes, and to cause death of eukaryotic cells. T–2 toxin has a direct lytic effect on erythrocytes [85].

#### Toxicity

T–2-contaminated products can cause severe effects in humans/animals at the same time it may even result in death [86, 87]. General signs of T-2 include nausea, emesis, dizziness, chills, abdominal pain, diarrhea, dermal necrosis, abortion, irreversible damage to the bone marrow, reduction in white blood cells (aleukia), inhibition of protein synthesis, and is toxic for the hematological and lymphatic systems, producing immunosupression [81, 87, 88]. The immune system is the mainly target of T–2, and the effects include changes in leukocyte counts, delayed hypersensitivity, depletion of selective blood cell progenitors, depressed antibody formation, allograft rejection, and a blastogenic response to lectins [5] and cytotoxic effect in cell culture [86]. T–2 alters the levels of dopamine, tryptophan, serotonine and serotonine metabolites in the brain of rodents and pigs [63]. T–2 is theoretically associated with a disease of past significance in Russia known as ATA [62]. The symptoms of illness included nausea, vomiting, necrotic lesions in the mouth and throat (making it difficult to eat), hemorrhaging in many of the body organs. Pacin *et al.* [88] reported T–2 was found increase in the level of monocytes in old mice, so this could be a biological indicator for T–2 subclinical intoxication.

The first human toxicosis in China due to moldy rice contaminated with *Fusarium* and T-2 toxin was reported by Wang *et al.* [89]. T–2 decreased responses to mitogens in human lymphocytes *in vitro*. No *in vivo* data from humans with known exposures are available, but effects on lymphocytes were recorded in Russia in persons affected by the ATA, an epidemic where T–2 is suspected to be causative agent [84]. The Committee noted that IARC (1993) concluded that no data were available on the carcinogenicity to humans of toxins derived from *F. sporotrichioides* and *F. sporotrichioides* are not classifiable as to their carcinogenicity to humans (Group 3). There is limited evidence in experimental animals for the carcinogenicity of T-2 [84].

### 3.2. DON

#### Short history and synthesis

DON is called as vomitoxin because of its strong emetic effects and its action as a feed refusal factor and it was first characterized and named following its isolation from *Fusarium*-infected barley in Japan. DON is produced by *F. graminearum* and *F. culmorum* among other *Fusarium* species [63, 64, 67]. Both species have different optimum temperatures for growth (25 and 21 °C, respectively) and this probably affects geographical distribution [87]. In developed countries where grains are dried to ≤13% moisture content to prevent mold growth, DON is the most important pre-harvest problem. However, it can also be produced during storage in the world where moisture content of stored grains is less rigorously controlled. Concurrent fungal infections with DON production in the field are mainly dependent on weather conditions and are favored by low temperatures and high humidity [63].

#### Stability

Since DON can be found in many post-harvest products, it is mostly believed to be resistant to standard processes like milling, baking and heating. Several chemical reagents such as ammonia, calcium hydroxide, chlorine, hydrochloric acid, ozone, sodium bisulfite and sodium hydroxide can degrade DON; however, to date none have been applied because these chemicals interfere with standard processing of grains or represent health hazards on their own [63]. DON is stable under weakly acidic conditions but is unstable under alkaline conditions like those encountered during tortilla preparation. A 72–88% decrease in natural corn contamination with DON has been shown during this process [74]. Naturally contaminated flour (1.200 μg/kg) and artificially contaminated flour (260 μg/kg) were used to prepare turnover pie dough covers. Frying was carried out at three temperatures (169°C, 205°C and 243°C) for different times. The final time for cooking at every temperature was accepted by measuring the colour during the frying process. DON reduction was greater in contaminated flour artificially with DON (>66% at 169°C, 43% at 205°C and 38% at 243°C). For the level of 1.200 μg/kg, the average percentage of DON reduction, based on medians, was 28% when the dough covers were fried at 169°C, 21% at 205°C and 20% at 243°C [90].

#### Products

DON is a mycotoxin that commonly contaminates cereal-based foods worldwide. It is detected often at the ppm level [63, 70]. DON is generally found in various cereal crops such as wheat, barley, oats, rye, rice and corn and is produced mainly by two important cereal pathogens: *F. graminearum* Schwabe and *F. culmorum* Sacc., which cause ear rot in maize and head blight in wheat [5, 87, 91]. Natural occurrence of DON in cereals is certainly prevalent and surveys from South America, Canada, China and many countries of Europe have showed contamination levels in excess of 50% in oats, barley and wheat with mean concentrations as high as 9 mg/kg in barley [87]. DON and either of two mono-acetylated derivatives – 3-and 15-acetyl DON – are frequently found together in cereal-based products [67, 92]. DON is the most often occurring TC and is prevalent in crops used for food and feed production [67, 93].

#### Mode of action

The molecular mode of action of DON involves disruption of normal cell structure and function by inhibiting protein synthesis via binding to the ribosome and by activating critical cellular kinases involved in signal transduction related to proliferation, differentiation, and apoptosis [63]. The mechanism of these effects has not been fully explained but alters in the serotonergic activity of both the peripherous and central nervous system may be involved [67].

#### Toxicity

At the cellular level, the main toxic effects of DON are immunosuppressant or immunostimulation depending upon the dose and duration of exposure. Although these effects have been largely characterized in the mouse, several investigations with DON suggest that immunotoxic effects are also likely in domestic animals [94]. The symptoms of acute toxicity studies in sensitive species include abdominal distress, increased salivation, malaise, diarrhea, emesis and anorexia. In addition to, the most common effects of chronic toxicity studies in experimental animals are decreased weight gain, anorexia, and altered nutritional efficiency [63].

The main effects of DON at low dietary doses appear to be decreased growth and anorexia, while higher doses induce vomiting (emesis), immunotoxic effects and changes in brain neurochemicals [93]. In animals, at low dosages of DON, hematological, clinical and immunological alterations are also temporary and decrease as compensatory/adaptation mechanisms are founded. According to the sensitivity between the species, pigs are more sensitive to DON than mice, poultry, and ruminants, in part because of differences in metabolism of DON, with males being more sensitive than females. [94]. For all that, all animal species tested have been shown to be susceptible to DON. Experimental animals are sensitive to DON according to the following rank order: swine>mice>rats>poultry>ruminants. Animal species differ with regard to the absorption, distribution, metabolism, and elimination of DON. This may account for the differential sensitivity to the adverse effects of this mycotoxin [62, 63].

DON is less toxic than other TCs such as T-2, however, highly DON doses (i.e. unlikely to be encountered in food) can cause shock-like death [70]. Studies with experimental animals demonstrated effects on the immune system [77, 95], neuroendocrine effects of DON [94], also, toxic effects of DON on human and animal have been reviewed in detail by [63].

From acute toxicity studies in animals it seems that DON might produce similar effects in humans [63]. *In vivo* DON suppresses normal immune response to pathogens and concurrently induces autoimmune-like effects which are similar to human immunoglobulin A (IgA) nephropathy [94]. There have been reports that in Asia of illness in humans, such as vomiting, nausea, dizziness and headaches, associated with the consumption of cereals contaminate with DON and possibly much lower doses of other TCs [87]. In 1993, IARC placed DON in Group 3, not classifiable as to its carcinogenicity to humans [5, 10]. A provisional maximum tolerable intake (PMTDI) of 1 μg/kg body weight (BW) was set by [63].

### 3.3. NIV

#### Short history and synthesis

Nivalenol (3,4,7,15-tetrahydroxy-12,13-epoxytrichothec-9-en-8-one) is one of the well-known mycotoxins among naturally occurring TCs. Based on a survey conducted from 1976 to 1985, TCs, especially NIV was detected in Japanese wheat and barley grains [96]. NIV may generally occur together with fusarenon X. *Fusarium cerealis* and *F. poae* are the main producers of NIV, but isolates of *F. culmorum* and *F. graminearum* are also able to produce nivalenol. *F. poae* is reported the main producer of nivalenol in Sweden [64]. *F. poae* is more widespread in Europe and an important producer of NIV [97]. Owing to its high storage stability and food processing, it resists to high temperatures [98].

#### Stability

NIV occurs more often in years with dry and warm growing seasons. NIV is more frequently reported in Europe, Australia and Asia than in America. Both mean levels and incidence of positive samples of NIV are lower than for DON even in the Nordic countries and Europe [64].

#### Products

NIV occurs in various cereal crops such as wheat, corn, barley, oats, and rye [98, 99]. It has also been frequently detected in cereal grains and foods produced in Korea, China and other countries, and are thought to induce several food-borne diseases [96].

#### The mode of action

NIV is a potent inhibitor of protein, RNA, DNA synthesis in mammalian cells and causes necrosis of the proliferating cells *in vivo* [96]. Because of this, NIV is especially toxic to rapidly dividing tissues [100] such as lymphoid organs and intestinal mucosa [98]. NIV induces apoptosis in HL60 cells [101].

#### Toxicity

In mice, NIV is embryotoxic and fetotoxic but not teratogenic [102]. NIV inhibits Ig production in mice [103]. NIV slightly increased the frequencies of chromosomal aberrations and sister chromatid exchange (SCE) in Chinese hamster cells [99]. NIV is a weak inducer of chromosomal aberrations in mammalian cells *in vitro* and from tests it seems that NIV has the possible to cause DNA-damage. However, the available information is too limited (a.o.no gene mutation tests) to evaluate the genotoxic potential of NIV [102]. Acute/chronic toxicity results showed that 6 ppm or more of ingestion NIV for one year exhibit a characteristic toxic effect of NIV in mice. Acute NIV induces bone marrow toxicity. Chronic toxicity exposure may also cause leucopenia [104]. There is insufficient evident of carcinogenicity of NIV in experimental animals. Also, no human data were available [10, 99].

### 3.4. DAS

#### Short history and synthesis

DAS (3-hydroxy-4,15-diacetoxy-12,13-epoxytrichothec-9-ene) is produced by certain *Fusarium* species such as *F. poae, F. semitectum, F. verticillioides, F. sporotrichioides, F. acuminatum, F. culmorum, F. crookwellense, F. venenotum, F. sambucinum, F. equiseti, F. graminearum, F. avenaceum, F. langsethiae* [105, 106]. DAS belongs to a group of mycotoxins called the 12,13-epoxytrichothecenes which consist of closely related sesquiterpenoids produced by several imperfect fungi. DAS was first isolated from cultures of *F. scirpi, F. equiseti* and *Gibberella intricans* [107].

#### Stability

It is observed that DAS increased at low temperatures in corn and rice. Activity of water (a_w_) is an important parameter for toxin production in corn and wheat. An optimal a_w_ in rice is found as 0.995 [108, 109].

#### Products

DAS is one of the TC mycotoxins naturally occurring in agricultural products [110, 111]. DAS is abundant in various cereal crops such as corn, barley, mixed feed samples and other grains from various regions in the world. Co-existence of DAS and T-2 in animal feeds and human foods represent a health threat to humans and animals in some parts of the world [110].

#### Mode of action

DAS was rapidly transformed to four products, including 15-monoacetoxyscirpenol (MAS), scirpenol and two new compounds identified as 15-acetoxy-3α,4β-dihydroxytrichothec-9,12-diene(deepoxy MAS) and 3α,4β,15-trihydroxytrichothec-9,12-diene (deepoxyscirpentriol) [112].

#### Toxicity

This mycotoxin shows a wide-ranging biological activity, including toxicity to fungi, plants, animals, and various mammalian tissue cultures [105]. The order of sensitivity to DAS was swine>dogs>>cattle [10, 102]. Toxic effects of DAS in humans and animals seemed similar such as nausea, vomiting, diarrhea, hypotension, neurological symptoms, chills and fever. Also, the hematopoietic system appeared extremely sensitive, showing severe myelosuppression. In animals these symptoms were independent of the route of dosing [102, 113]. DAS is found as teratogen in mouse [114]. Thuvander *et al.* [77] found that DAS effectively inhibited proliferation and Ig production in mitogen-stimulated human lymphocytes in a dose-dependent manner with limited variation in sensitivity between individuals. DAS was found as esophageal hyperplasia but not cancer in rat [115]. DAS has undergone clinical trials as a chemotherapeutic agent in cancer patients [102].

### 3.5. FUS-X

#### Short history and synthesis

FUS-X, a type B TC, was first isolated in 1968, and characterized in 1969. It is not mentioned very frequently in the research, as the main interest for TCs concerns DON, T-2 and HT-2 [93]. FUS-X is one of the TC mycotoxins isolated from *Fusarium nivale* [116]. FUS-X is produced by different species of the genus *F. graminearum*, *F. oxysporum*, *F. semitectum*, *F. sporotrichioides*, *F. sambucinum* [117].

#### Stability

FUS-X might occur more frequently in the warmer and subtropical part of the world [117].

#### Products

It may occur such as garlic, corn, oats, and wheat [117].

#### Mode of action

FUS-X belongs to a group of sesquiterpenoid mycotoxins, chemically classified as 12,13-epoxytrichothecenes, which are potent and selective inhibitors of protein synthesis in eukaryotes [118]. FUS-X has been found to inhibit not only protein but also DNA synthesis, while RNA synthesis was not or only marginally affected [102]. FUS-X is particularly potent inhibitor of protein synthesis and interferes primarily with high division rate tissues such as spleen, bone marrow, thymus and intestinal mucosa [119]. FUS-X binds to the peptidyltransferase catalytic center on ribosomes and blocks the extension of the peptide chain [120]. FUS-X is rapidly deacetylated to NIV *in vivo* and frequently co-occurs with NIV in amounts corresponding to 10%–20% of the amount of NIV present [121].

#### Toxicity

FUS-X is immunosuppressive, carcinogenic, cytotoxic, emetic, and causes diarrhea, hypothermia, decreased respiratory rate in experimental animals [62]. FUS-X is highly cytotoxic to cultured cells [102]. Like other TCs, FUS-X inhibits lymphocyte blastogenesis. FUS-X is toxic to murine thymocytes, lymphocytes, and gastric epithelial cells and human hepatoblastoma cells. The effects to thymocytes and lymphocytes can be classified as immunotoxic [93]. FUS-X is known to be potent inducer of apoptosis in mouse thymocytes both *in vivo* and *in vitro* [119, 120].

IARC reported that inadequate information was available for determining the carcinogenicity for experimental animals [93]. FUS-X induced non-lymphocytic suppressor cells in the spleen of the treated mice [116].

No epidemiological data were available to assess the carcinogenicity of FUS-X for humans. Although there is a risk of exposure to FUS-X by consumption of contaminated food no data are available to evaluate the teratogenicity or chromosomal effects of FUS-X in humans [93]. FUS-X is known to be cytotoxic to many kinds of mammalian cells [118, 122]. Bony *et al.* [119] showed that the existance of a genotoxic potential for FUS-X at low exposure levels.

IARC [10] concluded that there is inadequate evidence in experimental animals for the carcinogenicity of FUS-X. No human data are available, and overall IARC concluded that FUS-X is not classifiable as to its carcinogenicity to humans (Group 3).

## 4. ZEA

### Short history and synthesis

ZEA was discovered as the cause of a reproductive disorder in pigs known as vulvovaginitis [87]. It is one of the most common *Fusarium* mycotoxins in the temperate regions of America, Europe and Asia. It is most frequently encountered on corn, but also contaminates other cereals and plant products [123]. ZEA (previously known as F-2 toxin) may occur in the form of four hydroxyl derivatives [124]. ZEA is a non steroidal, estrogenic mycotoxin produce by *Fusarium* species [125, 126]. ZEA and zearalenol are produced by the fungi *Fusarium* spp. Mycotoxins produced by *Fusarium* spp. are of two general types: 1) the nonestrogenic TCs, including DON, NIV, T-2, and DAS; 2) the mycoestrogens, including ZEA and zearalenol [127]. ZEA and some of its metabolites have been shown to competitively bind to estrogen receptors. The relative binding affinities to the rat uterine cytoplasmic receptor for ZEA and derivatives are α-zearalenol>β-zearalanol>ZEA>β-zearalenol, respectively [5]. The most important characteristic of *Fusarium* species is their ability to synthesize ZEA, and its co-occurrence with certain TCs raises important point regarding additive and/or synergism in the etiology of mycotoxicoses in animals [124].

### Stability

ZEA is a stable compound both during storage/milling and the processing/cooking of food. It does not degrade at high temperatures. Wet milling of corn levels ZEA in the gluten fraction (2–7 fold concentration) [126].

### Products

ZEA is found, especially, as a contaminant in corn. Also, it may occur in oats, barley, wheat and sorghum [75, 125] as shown in Table 5. However, ZEA production is favored by high humidity and low temperatures conditions [62]. It may co-occur with DON in grains such as wheat, barley, oats and corn and fumonisins in corn [128]. Sometimes ZEA may occur as a contaminant co-exist DON. Generally, DON is found in higher doses than ZEA when this occurs [75].

### The mode of action

The mode of action of ZEA and its derivatives involves displacement of estradiol from its uterine binding protein, elucidating an estrogenic response [62, 126].

### Toxicity

It is associated with reproductive problems in specific animals and possibly in humans [125]. *In vivo* studies have showed that ZEA is rapidly metabolized in animals and humans. Free and conjugated forms of ZEA have been found in the milk of cows under experimental conditions. That high concentrations of the toxin are required to elicit such a response indicates that consumption of contaminated feed by dairy cows would not result in a risk to public health [125]. ZEA at amounts greater than normally encountered in field exposures (200 mg/kg of feed) does not affect adversely the reproductive potential of mature boars [75, 129]. ZEA and some of its metabolites have been shown to competitively bind to estrogen receptors in a number of *in vitro* systems. Bindings to specific receptors have been displayed in uterus, mammary gland, liver and hypothalamus in different species [126].

In any event, the contamination of corn with ZEA is a threat to animal and public health and seriously reduces the quality of corn products. Fertility problems have been observed in animals such as swine and sheep [123]. Any compound with hormonal activity may be genotoxic and/or carcinogenic and there is some case that ZEA may show both types of activity in some animal species [87]. ZEA can be transmitted to piglets in sows’ milk, causing estrogenism in pigs. The most important effects of ZEA primarily include the urogenital system. Swine are the most commonly affected animals. Also, cattle, poultry and laboratory rodents affected [62]. ZEA causes changes in the reproductive system of laboratory animals such as mice, rats, guinea-pigs, hamsters, rabbits and domestic animals [126]. ZEA may be an important etiologic agent of intoxication in young children or fetuses exposed to this mycotoxin, which results in premature thelarche, pubarche, and breast enlargement [62]. ZEA was evaluated by the International Agency or Research on Cancer in 1993, based on inadequate data in humans and limited evidence in experimental animals ZEA was allocated, together with other *Fusarium* toxins, in group 3 (not classifiable as to their carcinogenicity to humans) (Table 3). Hepatocellular adenomas and pituitary tumors were observed in carcinogenicity in mice [126].

Because of its hormonal activity there is considerable knowledge about ZEA and its derivatives to be found in the patent literature on growth hormones as there is in the literature on mycotoxins. Its use for increasing meat production in cattle is allowed in some countries, such as the USA, and forbidden in others, such as the countries of the European Community. Such differences in legislation in different parts of the world cause to difficulties in trade between such countries [87]. FDA recommends that animals implanted with this agent must be kept from slaughter for at least 60 days and post implantation were not monitored since farmers are not aware of the hormonal product [62].

## 5. Conclusions

Between 1987 and 2002, the studies of DON in cereal samples (wheat, barley, oat, rye) collected Germany have been reported by Goyarts [130]. In 1999, the worldwide contamination of *Fusarium* mycotoxins (DON, NIV, ZEA, DAS, T-2, HT-2) in cereal grains have been reported by Placinta *et al.* [124]. In 2000, mycotoxin contamination (DON, NIV, ZEA) in rice have been suggested by Tanaka *et al.* [131]. In 2001 the SCOOP (Scientific Co-operation on Questions relating to Food) have been reported data of *Fusarium* toxins (DON, NIV, FUS-X, T-2, HT-2, DAS, ZEA) in cereals (wheat, corn, barley, oat, rye) collected from 12 countries (The Netherlands, Norway, Portugal, Sweden, UK, Italy, Germany, France, Finland, Denmark, Belgium, Austria) [132]. Between 2003 and 2005, the studies of DON, T-2 toxin, ZEA and fumonisins (FB_1_+FB_2_+FB_3_) in cereal samples collected from European and Mediterranean markets and Asian-Pacific region have been reported by Binder *et al* [133].

The limit values of *Fusarium* mycotoxins in cereal and cereal products (in the USA, EU and Turkey) are given by [4, 134–137].

Cereal products are important in our food chain and economy. Therefore, foodstuffs need to be controlled/analyzed during food processing and all mycotoxin analyses for the entire food chain has importance for human health. It is important to continue to monitor the occurrence of these mycotoxins in cereals and cereal products.

## Figures and Tables

**Table 1.** Carcinogenicity risk evaluated by IARC^a^ for *Fusarium* mycotoxins.

Toxins	Degree of evidence of carcinogenicity^a^		Overall evaluation^a^
	In humans	In animals	
Toxins derived from:			
*F. graminearum,*			
*F. culmorum,*			
*F. crookwellense*	I		Group 3
ZEA	ND	L	
NIV		I	
FUS-X		I	
DON		I	
Toxins derived from:			
*F. sporotrichioides*	ND		Group 3
T-2		L	
Toxins derived from:			
*F. verticillioides*	I	S	Group 2B
FB_1_		L	
FB_2_		I	
Fusarin C		L	

**^a^** **I:** insufficient evidence; **L:** limited evidence; **ND:** no adequate data; **S**: sufficient evidence; **Group 2B:** possibly carcinogenic to humans; **Group 3**: not classifiable as to its carcinogenicity to humans. *From IARC, 2003.

**Table 2.** World-wide occurrence of fumonisins in cereals.

Product	Detected/total	Range	Country	References
	**FB_1_**: 22/110	**FB_1_**: nd–2.66 ppm		
Corn			Turkey	6
	**FB_2_**: 0/110	**FB_2_**: nd		
Corn	**TFBs:** 30/92	**TFBs:** 0.3–273 mg/kg	Turkey	138
Corn	**FB_1_**: 10/20	**FB_1_**: 10–5960 μg/kg	Morocco	139
Corn	**FB_1_**: 19/57	**FB_1_**: 10–780 μg/kg	Egypt	140
	**FB_1_**: 6.99/60	**FB_1_**: 127–359 ng/g		
Corn			Spain	141
	**FB_2_**: 5.01/60	**FB_2_**: 60–153 ng/g		
Corn	**FB_1_**: 18/27	**FB_1_**: 48–918 mg/L	Croatia	142
Corn	**FB_1_**: 80/184	**FB_1_**: 0.21–3.30 μg/g	China	14
Corn	**TFBs:** 24/31	**TFBs:** nd–34700 μg/kg	Argentina	143
Corn	**FB_1_**: 10/10	**FB_1_**: 0.3–1.5 mg/kg	Côte d’Ivoire	144
	**FB_1_**: 49/49	**FB_1_**: 142.2–1377.6 μg/kg		
Corn			Croatia	145
	**FB_2_**: 3/49	**FB_2_**: 68.4–3084.0 μg/kg		
Corn	**TFBs:** 96/196	**TFBs:** 2242 μg/kg (max)	Brazil	146
Corn	**FB:** 0/6	**FB:** nd	Canada	147
Corn	**FB:** 41/42	**FB:** 0.012–0.84 ppm	Argentina	148
	**FB_1_**: 0/19	**FB_1_**: nd		
Wheat			Turkey	6
	**FB_2_**: 0/19	**FB_2_**: nd		
Wheat	**FB_1_**: 4/210	**FB_1_**: 1–2 ppm	S. Africa	149
Barley	**FB:** 0/10	**FB:** nd	Canada	147
	**FB_1_**: 0/1	**FB_1_**: nd		
Oat			Turkey	6
	**FB_2_**: 0/1	**FB_2_**: nd		
Oat	**FB:** 0/5	**FB:** nd	Canada	147
	**FB_1_**: 0/31	**FB_1_**: nd		
Rice			Turkey	6
	**FB_2_**: 0/31	**FB_2_**: nd		
Rice	**FB_1_**: 0/10	**FB_1_**: nd	Côte d’Ivoire	144
Rice	**FB_1_**: 2/88	**FB_1_**: 48.2–60.6 ng/g	Korea	150
Rice	**FB:** 1/25	**FB:** 10 ng/g	Canada	147
	**FB_1_**: 52.52/202	**FB_1_**: 0.010–2.870 μg/g		
Cereal			Italy	151
	**FB_2_**: 70.7/202	**FB_2_**: 0.010–0.790 μg/g		
Cereal	**FBs:** 30.08/32	**FBs:** 1–1110 μg/kg	France	152

**TFBs**: Total fumonisins

**Table 3.** Human Foods.

Products	Total Fumonisins(FB_1_ + FB_2_ + FB_3_) (ppm)
Degermed dry milled corn products (e.g., flaking grits, corn grits, corn meal, corn flour with fat content of < 2.25 %, dry weight basis)	2
Whole or partially degermed dry milled corn products (e.g., flaking grits, corn grits, corn meal, corn flour with fat content of ≥2.25 %, dry weight basis)	4
Dry milled corn bran	4
Cleaned corn intended for masa production	4
Cleaned corn intended for popcorn	3

**Table 4.** Some national estimates of intake of FB_1_ in Europe and in the world.

Country	Intake (μg/kg of body weight per day)	
	Mean or median	90th percentile
Argentina	0.2	nd
Canada	0.02	0.08
Netherlands	0.06, 1.0^a^	nd
Switzerland	0.03	nd
United Kingdom	0.03	0.1
United States	0.08	nd

**nd:** not reported or calculated.

**^a^** The first value is for whole population, the second for regular maize eaters.

**Table 5.** World-wide occurrence of trichothecenes and zearalenone in cereals.

Product	Detected/total	Range	Countries	References
	**DON:** 0/38	**DON:** nd		
Corn			Argentina	153
	**ZEA:** 0/38	**ZEA:** nd		
	**DON:** 0/78	**DON:** nd–60 ng/g		
	**NIV:** 0/78	**NIV:** nd–40 ng/g		
Corn	**DAS:** 0/78	**DAS:** 20–120 ng/g	Brazil	154
	**T-2:** 1/78	**T-2:** 20–100 ng/g		
	**HT-2:** 1/78	**HT-2:** 20–100 ng/g		
	**DON:** 3/4	**DON:** 0.065 μg/g		
Corn			Poland	155
	**NIV:** 3/4	**NIV:** 0.13 μg/g		
	**DON**: 8/40	**DON:** 204–745 μg/kg		
Corn			Nigeria	156
	**DAS:** 1.5/17	**DAS:** 23–51 μg/kg		
	**NIV:** 6/31	**NIV:** 12–2440 ng/g		
	**DON:** 6/31	**DON:** 5–2060 ng/g		
Corn			Italy (Central Italy)	157
	**FUS-X:** 5/31	**FUS-X:** 26–420 ng/g		
	**ZEA:** 5/31	**ZEA:** nd–384 ng/g		
	**NIV:** 1/11	**NIV:** 200 ng/g		
	**DON:** 11/11	**DON:** 45–3430 ng/g	Italy (North-western Italy)	
Corn				157
	**FUS-X:** 1/11	**FUS-X:** 34 ng/g		
	**ZEA:** 17/11	**ZEA:** nd–969 ng/g		
	**NIV:** 0/4	**NIV:** nd		
	**DON:** 3/4	**DON:** 68–967 ng/g		
Corn			Italy (Po valley)	157
	**FUS-X**: 0/4	**FUS-X:** nd		
	**ZEA:** 0/4	**ZEA:** nd		
	**DON:** 4/21	**DON:** nd- 834.4 μg/kg		
Corn			Argentina	143
	**ZEA:** 11/23	**ZEA:** nd- 2564.8 μg/kg		
Corn	**T-2:** 8/30	**T-2:** 0.45–1.70 ppm	Turkey	81
	**DON**: 47/175	**DON:** 26.1–131.7 μg/kg		
Corn			Spain	158
	**NIV:** 7/175	**NIV:** 51.1–106.5 μg/kg		
Corn	**ZEA:** 3/20	**ZEA:** 12–17 μg/kg	Morocco	139
Corn	**TTCs:** 42/196	**TTCs:** <2–600 μg/kg	Saudi Arabia	159
Corn	**ZEA:** 10/10	**ZEA:** 20–50 μg/kg	Côte d’Ivoire	144
Corn	**ZEA:** 41/49	**ZEA:** 0.9–2.54 μg/kg	Croatia	145
	**DON:** 0/6	**DON:** nd		
Corn	**ZEA:** 0/6	**ZEA:** nd	Canada	147
	**NIV-HT-2**: 0/6	**NIV-HT-2:** nd		
	**DON:** 58.8/60	**DON:** 15–1379 μg/kg		
	**NIV:** 7.2/60	**NIV:** 25–40 μg/kg		
	**HT-2:** 4.2/60	**HT-2:** 12 μg/kg		
Wheat			Germany	160
	**T-2:** 1.2/60	**T-2:** 4 μg/kg		
	**ZEA:** 22.8/60	**ZEA:** 1–24 μg/kg		
	**FUS-X:** 0/60	**FUS-X:** 0		
	**DON:** 108/121	**DON:** 10–2591 μg/kg		
	**NIV:** 57/120	**NIV:** 10–234 μg/kg		
Wheat	**HT-2:** 6/36	**HT-2:** nd–33 μg/kg	Denmark	161
	**T-2:** 11/38	**T-2:** nd–153 μg/kg		
	**ZEA:** 10/30	**ZEA:** 1–2 μg/kg		
Wheat	**DON:** 8/12	**DON:** 0.01 μg/g	Poland	155
	**DON:** 144/150	**DON:** Tr-642 μg/kg		
Wheat			Lithuania	162
	**ZEA:** 32/100	**ZEA:** Tr-95.6 μg/kg		
Wheat	**T-2:** 0/3	**T-2:** nd	Turkey	81
Wheat	**TTCs:** 0/1	**TTCs:** nd	Saudi Arabia	159
	**DON:** 23.17/169	**DON:** 350 μg/kg (max)		
	**NIV:** 0/169	**NIV:** 10 μg/kg (max)		
Wheat			Norway	163
	**HT-2:** 1.86/169	**HT-2:** 20 μg/kg (max)		
	**T-2:** 1.24/169	**T-2:** 20 μg/kg (max)		
Wheat	**ZEA:** 22/24	**ZEA:** 11–860 μg/kg	Germany	164
	**DON:** 10/10	**DON:** 0.025 μg/g		
Barley			Poland	155
	**NIV:** 10/10	**NIV:** 0.04 μg/g		
	**DON:** 53/55	**DON:** Tr-372 μg/kg		
Barley			Lithuania	162
	**ZEA:** 38/66	**ZEA:** Tr-193.4 μg/kg		
Barley	**DON:** 157.45/292	**DON:** 500–10.000 μg/kg	Uruguay	165
Barley	**TTCs**: 8/93	**TTCs:** 3.1–4.000 μg/kg	Saudi Arabia	159
	**DON:** 17.6/102	**DON:** 1440μg/kg (max)		
	**NIV:** 11.2/102	**NIV:** 50 μg/kg (max)		
Barley			Norway	163
	**HT-2:** 39.78/102	**HT-2:** 440 μg/kg (max)		
	**T-2:** 8.16/102	**T-2:** 220 μg/kg (max)		
Barley	**DON-NIV:** 41/84	**DON-NIV:** 40–2340 μg/kg	Ethiopia	166
	**DON:** 29/50	**DON:** 980 ng/g (max)		
Barley	**ZEA:** 4/29	**ZEA:** 22 ng/g (max)	Canada	147
	**NIV-HT-2:** 0/75	**NIV-HT-2:** nd		
	**HT-2:**24/99	**HT-2:** 10–47 μg/kg		
Oats	**T-2:**15/99	**T-2:** 10–703 μg/kg	Poland	167
	**ZEA:**19/99	**ZEA:** 10–118 μg/kg		
	**DON:** 12/12	**DON:** 0.027 μg/g		
Oats			Poland	167
	**NIV:** 12/12	**NIV:** 0.033 μg/g		
	**DON:** 13/14	**DON:** Tr-204 μg/kg		
Oats	**ZEA:** 4/7	**ZEA:** Tr-16.3 μg/kg	Lithuania	162
	**T-2:** 10/10	**T-2:** 10.8–121.5 μg/kg		
Oats	**T-2:** 0/1	**T-2:** nd	Turkey	81
	**HT-2:** 24/99	**HT-2:** 10–47 μg/kg		
Oats	**T-2:** 15/99	**T-2:** 10–703 μg/kg	Poland	167
	**DAS:** 12/99	**DAS:** 10–118 μg/kg		
Oats	**TTCs**: 0/3	**TTCs**:nd	Saudi Arabia	159
	**DON:** 101.96/178	**DON:** 849 μg/kg (max)		
	**NIV:** 17.6/178	**NIV:** 211 μg/kg (max)		
Oats			Norway	163
	**HT-2:** 124.04/178	**HT-2:** 880 μg/kg (max)		
	**T-2:** 53.32/178	**T-2:** 380 μg/kg (max)		
	**DON:** 33/53	**DON:** 90 ng/g (max)		
Oats	**ZEA:** 0/6	**ZEA:** nd	Canada	147
	**NIV-HT-2**: 0/53-0/53	**NIV-HT-2:** nd		
	**DON:** 10/10	**DON:** 0.023 μg/g		
Rye			Poland	155
	**NIV:** 5/10	**NIV:** 0.002 μg/g		
	**DON:** 14/16	**DON:** Tr-691 μg/kg		
Rye			Lithuania	162
	**ZEA:** 0/10	**ZEA:** 0–28.8 μg/kg		
	**DON**: 41/69	**DON:** 10–257 μg/kg		
	**NIV:** 9/69	**NIV:** nd–48 μg/kg		
Rye	**HT-2:** 11/26	**HT-2:** 10–70 μg/kg	Denmark	161
	**T-2:** 12/25	**T-2:** nd–193 μg/kg		
	**ZEA**: 2/30	**ZEA:** 1–2 μg/kg		
	**DON**: 3/88	**DON:** 105– 159 ng/g		
Rice	**NIV:** 5/88	**NIV:** 182– 462 ng/g	Korea	150
	**ZEA**: 3/88	**ZEA:** 21.7– 47.0 ng/g		
Rice	**TTCs:** 0/2	**TTCs:** nd	Saudi Arabia	159
Rice	**ZEA**: 10/10	**ZEA:** 50–200 μg/kg	Côte d’Ivoire	144
	**DON**: 0/9	**DON:** nd		
Rice	**ZEA:** 1/7	**ZEA:** 1 ng/g (max)	Canada	147
	**NIV-HT-2:** 0/27	**NIV-HT-2:** nd		
Cereals	**DAS**: 0/69	**DAS:** nd- 0.8 μg/g	Turkey	106
Cereals	**DON:** 169.68/202	**DON:** 0.007–0930 μg/g	Italy	151
Cereals	**T-2:** 2/30	**T-2:** 1.60–33.30 ppm	Turkey	168
	**DON:** 12/12	**DON:** 16–51 450 μg/kg		
Cereals	**NIV:** 10/12	**NIV:** 10–6935 μg/kg	China	169
	**ZEA:** 10/12	**ZEA:** 46–3079 μg/kg		
	**DON:**54/68	**DON:** 5–111 μg/kg		
Cereals	**NIV:**3/68	**NIV:** 10–20 μg/kg	Finland	170
	**HT-2**:2/68	**HT-2:** 10–20 μg/kg		
	**DON:** 126/169	**DON:** 15–1670 μg/kg		
	**NIV:** 0.21/4	**NIV:** 25–231 μg/kg		
Cereals	**FUS-X:** 0	**FUS-X:** nd	Germany	171
	**HT2:** 11.38/43	**HT-2:** 12–51 μg/kg		
	**T-2:** 1.43/10	**T-2:** 4–39 μg/kg		

**Tr:** traces; **nd:** not detected; **TTCs**: Total trichothecenes.

## Abbreviations:

FB: Fumonisin
TCs: Trichothecenes
DON: Deoxynivalenol
NIV: Nivalenol
DAS: Diacetoxyscirpenol
FUS-X: Fusarenone-X
HT-2: HT-2 toxin
T-2: T-2 toxin
ZEA: Zearalenone
